# Factors affecting compliance with use of online healthcare services among adults in Israel

**DOI:** 10.1186/s13584-016-0073-8

**Published:** 2016-06-15

**Authors:** Shosh Shahrabani, Yonathan Mizrachi

**Affiliations:** Department of Economics and Management, The Max Stern Yezreel Valley College, Emek Yezreel, 19300 Israel; Department of Sociology and Anthropology, The Max Stern Yezreel Valley College, Emek Yezreel, 19300 Israel

**Keywords:** Online health services, Compliance, Elderly

## Abstract

**Background:**

The use of online health services (henceforth, OHS) among middle-aged to older adults can make health-related actions more accessible to this population group as well as help reduce the burden on the health system and avoid unnecessary costs. The study’s objectives were to examine the responsiveness and willingness of individuals aged 45+ to use different OHS and to characterize the attitudes and main factors influencing that responsiveness.

**Methods:**

We conducted a telephone survey among a sample of 703 individuals constituting a representative sample of the Israeli population of individuals aged 45+. The research questionnaire integrates the principles of the Adopting Medical Information Technologies model and includes socio-demographic attributes.

**Results:**

The results indicate that 78 % of internet users claimed to use at least one OHS (79 % of the Jewish sector and 66 % of the non-Jewish sector). Nevertheless, 22 % of internet users do not use OHS. Most online use is on Health Maintenance Organizations (HMO) websites to obtain administrative information. Frequency of OHS use increases as the following factors increase: perceived ease of online use; extent of encouragement for online use; perceived reliability of online health services; and extent of advertisement exposure. The study found that OHS use is much more prevalent among wealthy populations. In addition, individuals’ attitudes and the extent of their exposure to advertisement influence their use and intention to use OHS.

**Conclusions:**

A number of recommendations emerge from the study: 1) For OHS use to increase online health websites should be made more accessible to middle aged-older adults individuals and those of different languages and cultures. 2) Programs should be developed to teach HMO staff to encourage patients to use OHS. 3) Media advertising that encourages the use of OHS should be expanded.

## Background

Today, a wide range of technological possibilities are available for online health-related actions, such as making doctor’s appointments via Health Maintenance Organizations (henceforth, HMO) websites, receiving medical examination results, searching for essential medical information online, and even administrating remotely Telemedicine services. The term Online Health Services (OHS) will be used here as a cover term for these services. Although the term OHS is broad and covers a diverse range of services (from administrative information to real-time telemedicine services, as will be explained later), the current article primarily focuses on the Consumer Health Informatics or Electronic Health Records—using information and communication systems to collect, analyze and distribute medical information—of OHS and less so on Telemedicine. The reasons for this preference are discussed later in this article. Yet despite the prominent advantages of these technologies, their use among older adults is relatively low. For example, the findings of a study among older adults in the United Stated [1] showed that the rate of use of Online Health Services (henceforth, OHS) was much lower among individuals aged 65+ compared to younger individuals. Furthermore, the rate of OHS use further drops, from 32 % among individuals aged 65–74 to 14.5 % among individuals aged 75–84 and to 4.9 % among individuals aged 85+. Additionally, in the United States the rate of OHS use was found to be higher among individuals with higher socioeconomic status [2].

Studies conducted in Europe show that the rate of internet use for OHS purposes varies greatly from country to country. A study conducted in seven European countries among a representative sample of the entire population (between the ages of 15–80) showed that this rate ranges from 23 % in Greece to 62 % in Denmark [3]. In Israel, only a third of the population uses the internet for health purposes. Moreover, internet users characterized by high online health literacy were younger, more educated and less inclined to be sick [4].

Recent research studies indicate that the use of online technologies in the health domain can significantly improve quality of life for older adults, enhance their access to health services and minimize increases in health costs [5–10]. Patients who use OHS reported feeling more comfortable and satisfied than before they started using OHS [11].

In this respect, the upcoming widespread adoption of Personal Health Record (PHR) systems for patients and consumers is likely to yield even higher levels of comfort and satisfaction among elderly populations in need of medical services and information, although some evidence from Israel does hint to some possible complications [12]. PHR is defined as: “A private, secure application through which an individual may access, manage, and share his or her health information. The PHR can include information that is entered by the consumer and/or data from other sources such as pharmacies, labs, and health care providers. The PHR may or may not include information from the electronic health record (EHR) that is maintained by the health care provider and is not synonymous with the EHR. PHR sponsors include vendors who may or may not charge a fee, health care organizations such as hospitals, health insurance companies, or employers” [13]. The intention of PHR is to provide a complete and accurate summary of an individual’s medical history which is accessible online and available locally with the patient [14]. Such information may go beyond simple static repositories for patient data and may combine data, knowledge, and software tools, which help patients to become active participants in their own care. When PHRs are integrated with EHR systems, they may provide even greater benefits for elderly populations. Hence, we see a potential triple synergy that is expected to develop and increase among OHS, PHR and EHR systems. As the future usage of OHS by elderly populations will grow (given that Digital Native populations will join the OHS ecosystems), so will market pressures (user demands) for more effective PHR and EHR systems. In turn, OHS systems will grow to provide an even improved value-proposition to their users. Thus, it is important to understand the factors influencing willingness to use OHS as well as the reasons for not using these services (barriers and inhibitors), as illustrated by the following examples.

Heart and Kalderon (2011) [15] examined two groups of older adults in the United States and Israel regarding their use of information and communication technologies, such as computers, internet and cell phones. The results showed that adoption of information technologies among older adults, while constantly growing, is still quite limited. The factors influencing use included age, marital status, education and health status. Furthermore, the results showed that older adults who perceived these technologies to be more effective tended to adopt them more.

Today OHS are becoming more diverse. Telecare, for example, uses technology along with clinical protocols for remote monitoring and supervision of patient health, enabling patients to remain in their own homes. Despite the expansion of this trend [16, 17] and despite its great effectiveness, worldwide responsiveness to such technologies is not sufficiently broad [18, 19]. The equipment is not considered user friendly and the benefits are not always sufficiently clear to the potential users [20].

Botsis and Hartvigsen [21] analyzed the use of Telecare among individuals with chronic diseases. Their results showed that the patients that participated in the survey were generally satisfied with home Telecare services, though they preferred combining home Telecare with conventional medical services. Furthermore, users mentioned that in most cases Telecare results in decreased costs because it saves time and eliminates the drive to the clinics. Nevertheless, despite the important benefits of home Telecare, many technical, organizational and ethical problems need to be solved before expanding its use.

To the best of our knowledge, comprehensive and up-to-date research examining extent of use and intention to use OHS and remote medical services has yet to be conducted among middle-aged to older adults in Israel. The current research examines the willingness of this group to use OHS and maps the main factors that influence the extent of OHS use and the intention to use these services in Israel.

### Theoretical Framework

The literature refers to several theoretical models for examining how information technologies are adopted. The two main models are: 1) TAM (Technology Acceptance Model [22, 23]), and 2) UTAUT (Unified Theory of Acceptance of Technology Model [24]). These technology adoption models have been implemented in different domains, including the health domain.

The original Technology Acceptance Model (TAM) was developed to describe patterns of adoption and use of new technologies, such as information systems. The model includes five main components. Its main assertion is that patterns of using technology systems in general and of information systems in particular, including medical information systems, will be particularly influenced by the following factors: a) perceived ease of use; b) perceived benefit of use; c) attitude towards system use; d) behavioral intention to use; e) actual system use. Research on this topic has shown that perceived benefit and perceived ease of use are the most important factors in the adoption of new technology systems, including information technologies. Additionally, studies in this domain indicated that perceived ease of use directly influences perceived benefit and that users’ attitudes toward system use directly influence behavioral intention to use the system [22].

Many studies adjusted the TAM model to information systems in the health domain [25–29]. Most of these studies discussed how employees in the health domain adopted information technologies, while only a few implemented the model among health system consumers who voluntarily use OHS [30–32].

Wilson & Lankton [32] implemented the TAM model among voluntary OHS consumers. In their study, they empirically examined the suitability and predictability of three theoretical models for adopting health information systems: the TAM model, the motivational model [33], and a model integrating the two other models. The study examined responsiveness to OHS use among a representative sample of 163 participants in the United States. The findings show that all three models accurately predict people’s intention to use OHS.

The UTAUT model [24] was formulated with the following four determinants of intention and usage: a. performance expectancy, the degree to which an individual believes that using the system will help her to attain gains in job performance, b. effort expectancy, the degree of ease associated with the use of the system, c. social influence, the degree to which an individual perceives that important others believe she should use the new system, and d. facilitating conditions the degree to which an individual believes that an organizational and technical infrastructure exists to support use of the system. UTAUT was empirically confirmed with data from two organizations.

Monkman and Kushniruk [34] introduced the Consumer Health Information System Adoption Model, which describes both consumer eHealth literacy skills and system demands on eHealth literacy as moderators with the potential to affect the strength of relationship between usefulness and usability (actual usage outcomes).

Huang [30, 35] developed a theoretical model called the Healthcare Information Adoption Model (hereinafter HIAM) that is based mainly on the TAM model and that integrates some parts of the Health Belief Model (HBM) as well [36, 37]. According to this model, the two mentioned models are in fact complementary models, so that integrating them can help explain and predict the adoption of medical information technologies as well as provide insights toward developing and setting policies for these technologies [35]. Huang [30] validated the HIAM model, which demonstrated high goodness of fit for predicting intention to use Telecare among patients in Taiwan. Furthermore, the findings of Huang’s research [35] show that in Taiwan, citizens’ intention to use Telecare technologies was significantly influenced by the perceived usefulness and perceived benefit of the Telecare technology.

In the current research, we use the validated HIAM model [30] in combination with parts of the integrated model of the TAM model and the motivational model as suggested by Wilson and Lankton [32]. We chose to integrate these models in this research because they are among the few models that refer to implementation of the theoretical framework of responsiveness to medical technology adoption in the case of consumers that voluntarily use OHS systems. Most of the other theoretical frameworks had been implemented among health systems employees. Additionally, the current research refers not only to the intention to use OHS (as in Huang’s research [30, 35]), but also to the extent of actual use of OHS in Israel today.

According to the theoretical framework we use, we predict that the perceived ease of use and perceived benefit categories together with the categories of perceived health threat, perceived barriers and external and internal motivations for action will all influence individuals’ attitudes towards the use of medical information systems. Accordingly, these will influence the intention to use available OHS and the extent of their actual use. We also predict that actual use of OHS will affect the extent of intention to use OHS in the next year. This hypothesis is based on previous findings that past experiences can alter people’s beliefs, coping strategies, and future behavior [38]. For example, past experience with influenza vaccine may shape individuals’ attitudes and beliefs toward the vaccine and in turn affect the intention to get the vaccine in the next year [39].

### Mapping the available health services in Israel

#### Definitions and domains

The professional literature contains several typologies and classifications of online health services or Consumer e-Health Applications (see especially Cabrera et al. [40]). The current study distinguishes two generic types of online health services: 1) Consumer Health Informatics or Electronic Health Records—using information and communication systems to collect, analyze and distribute medical information; 2) Telemedicine or TeleHealthcare—using information and communication systems that combine hardware components designated for surveillance, data analysis and remote treatment of patients. This research focuses on the first type of online health services, which are more common and advanced. At the same time, however, it maps attitudes and barriers with respect to the second type of online health services.

The current research focuses on three types of online health services as described below. Note that all of Israel’s main HMOs as well as most of the public hospitals already offer all the assorted services described below:

1. Formal administrative and content-related medical information (with a one-sided, or two two-sided interactive, formal and institutional emphasis) from medical institutions, such as appointment scheduling, lab test results, interactive guides and blogs; continuous mobile-based pregnancy surveillance.
2. Informal content-related medical information (with a two-sided, interactive, informal and non-institutional emphasis). This information comes from content-related websites, such as forums and medical information communities, independent blogs and blogs sponsored by pharmaceutical companies and private institutions.
3. Online medicine at home. such as monitoring systems that require designated hardware at home (for blood pressure, pulse and sugar level monitoring) that report back to treatment institute information systems via internet or mobile networks, for example by remotely activating and controlling designated appliances for chronic illnesses and geriatrics.

We examined the integrated theoretical model—the Health Information Adoption Model (HIAM)—with regard to the main groups of medical information technologies described above. To the best of our knowledge, no comprehensive and up-to-date research has examined the extent of use of online health services and remote medical services in Israel among the 45 and above age group. Such services will become more and more accessible to the Israeli public in the near future. The current research aims at examining this group’s willingness to use online health services and at understanding the main factors influencing extent of use and intention to use online health services in Israel.

More specifically, the objectives of the current research are: a) to examine the extent of use of diverse OHS and the intention to use these services among individuals aged 45+; b) to analyze and segment the extent of use and intention to use OHS by socio-demographic factors (e.g., Jews versus non- Jews) 1; c) to characterize the main factors influencing the extent of use and intention to use OHS, including attitudes and barriers, among individuals aged 45 + .

## Methods

### The sample

The telephone survey was conducted by a professional poll survey company among 703 participants, constituting a representative sample of the Israeli population of individuals aged 45+ from the Jewish and non-Jewish sectors^2^. The sampling error was 3.7 %. The interviews were conducted in Hebrew, Russian and Arabic. Data were collected during March 2014. The sample size was chosen according to distribution into sub-groups by socio-demographic variables and residence area (center of Israel and peripheral regions). More specifically, at the first stage the country was divided into 6 regions/clusters (including center of Israel and peripheral regions). In each region/cluster cities were sampled according to their relative proportion (from CBS data^3^). In each city/residence area families were randomly sampled. The filter question was: “Is there at least one individual in the household over the age of 45?”. The poll survey company contacted 2,510 households in Israel and the final sample included 703 participants: 569 individuals from the Jewish sector and 134 individuals from the non-Jewish sector. The overall response rate was approximately 28 %.^4^

### Research questionnaire

The research questionnaire, which appears in Appendix 1, included the following parts: 1) Personal details, including socioeconomic information, age, marital status, education, nationality, immigration year, religious identification (1 = very religious, 5 = secular), income (from 1 = much above average to 5 = much below average), place of residence, HMO membership, supplementary insurance, private insurance, employment status, and individual’s self-evaluation of health status. 2) Extent of digital literacy (web information search skills, web communication skills, such as e-mail use, and search engine use skills) (based on questionnaire by Mizrachi et al. [41]) and extent of digital health literacy (based on questionnaires by Lustria et al. [42], Andreassen et al. [3] and Choi [1]). 3) Questions for individuals who use the internet: frequency of OHS use, extent of intention to use OHS and patterns of use. 4) Questions concerning attitudes towards OHS, based on questionnaires by Huang [30] and Wilson and Lankton [32]. The HIAM variables included the following categories: perceived ease of use, perceived benefits, perceived health threat, perceived barriers for action, and external and internal motives for action. The possible responses for each sentence ranged from 1 = do not agree at all to 7 = agree to a large extent.

The questionnaire examined the main reasons for OHS use or non-use in Israel as well as the reasons for willingness or lack of willingness to use remote medical technologies. Among the reasons for lack of use were concern about online exposure of personal details and lack of expertise in website use. In the first stage, we administered a pilot questionnaire among approximately 50 individuals, and after making some improvements, we developed the final version of the questionnaire.

### Description of the statistical methods for data analysis

A chi-squared test (*χ*^2^) was used to determine the association between categorical variables, including personal factors, and dependent variables: a) frequency of OHS use and b) intention to use OHS in the following year. The statistical significance of the difference between the means of the continuous variables of the different groups was determined by one-way ANOVA (F test). Furthermore, a multiple linear regression was used to identify the influence of demographic factors, the HIAM categories and additional factors regarding extent of OHS use and the intention to use OHS.

## Results

### OHS use by socio-demographic and other variables

The telephone survey included 703 interviewees, 59 % of whom reported using the internet (use the internet via at least one of the following devices: computer, smartphone and tablet). Seventy-eight percent of internet users (constituting 46 % of the sample) stated that they use at least one OHS (meaning: HMO based administration information/HMO-based consultation/remote services). In the Jewish sector, 79 % of internet users use OHS, while in the non-Jewish sector 66 % reported using OHS. Most of the internet users (67 %) reported vising their HMO website to obtain administrative information, 45.6 % reported visiting forums to obtain medical information and only 17 % stated that they visit their HMO website to consult with a doctor.

Table 1 summarizes the distribution of the samples according to different characteristics. Furthermore, the table compares the percentage of users of each online service (among internet users) by the different variables.

**Table 1:** Sample distribution and OHS usage by socio-demographic and other variables

	All sample (*N*=703) %	Gender		Age			Religion		Education		Income		Marital status			HMO				Employment status	
		Women (*N =* 410)	Men (*N*=293)	45-59 (*N*=307)	60-69 (*N*=215)	70^a^ (*N*=181)	Jewish (*N*=579)	Other (*N*=124)	Less than 12 (*N*=125)	12 and more (*N*=575)	Under average (*N* = 311)	Average and more (*N =* 296)	Lives alone (*N*=126)	Lives with a partner (*N =* 238)	Lives with family (*N*=332)	Clalit (*N =* 442)	Macc-abi (*N*=136)	Leu-mit (N=53)	Meuh-edet (*N =* 68)	Works (*N*=317)	Do not work (*N*=385)
Sample distribution	100	58	42	44	30	26	82	18	18	82	51	49	18	34	48	63	20	7	10	45	55
Percentage of Internet users, out of sample^a^	59	53	56	63***	56	38	58***	36	14***	63	37***	71	49	53	58	48***	75	42	63	68***	43
Percentage of users of The HMO’s website for administrative information, out of Internet users^b^	67	69	64	64	67	75	70**	53	50*	68	58**	69	70**	75	61	45*	51	44	39	63**	72
Percentage of users of The HMO’s website for consultation, out of Internet users	17	18	16	17	18	15	16**	29	21	17	15	19	15	19	16	17	20	11	15	16	19
Percentage of users of Forums for medical information, out of Internet users	46	51**	39	48	42	44	47	39	29	46	39*	49	41	46	47	66	76	59	57	48	43

*** *p <* 0.01, ** *p <* 0.05, * *p <* 0.1

^a^ The statistical significance measures in row 3 the differences between percentages of Internet users by socio-demographic and other variable

^b^ The statistical significance measures in rows 4–6 the difference between percentages of users of each online service, out of Internet users, by variables

The findings in Table 1 indicate that among the internet users in the sample, the rate of individuals who visit *their HMO website to obtain administrative information* is significantly higher among Jews (69.5 %) than among non-Jews (52.9 %). This rate is also higher among individuals with 12+ years of education (67.9 %) than among individuals with less education (50.0 %), among individuals with an average income or higher (69.2 %) than among individuals with a less than average income (57.8 %), among individuals who do not work (72.2 %) than among those who work (62.9 %) and among individuals who live with a partner (75 %) than among individuals who live alone (69.7 %) and those who live with family (61.4 %). The rate of individuals who visit *their HMO website to consult with a doctor* was significantly higher among non-Jews (29.4 %) than among Jews (15.5 %). The rate of individuals who visit *forums to obtain medical information* was significantly higher among women (50.6 %) than among men (38.7 %), among native Israelis (50.0 %) than among others (40.1 %) and among individuals with an average or higher income (48.7 %) than among individuals with a lower than average income (39.1 %).

**Fig. 1:**
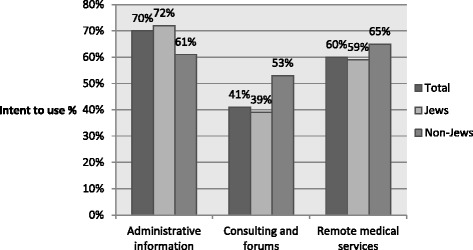
The percentage of internet users that intend to use OHS in the following year by type of service and religion

Table 2 and Fig. 1 show the percentage of internet users that intend to use OHS in the following year by type of service and religion.

**Table 2:** Median scores and percentage of individuals that intend to use the different types of OHS by religion^a^

Type of online service	Intention to use	Total	Jews	Non-Jews
HMO’s website for administrative information such as: Appointment scheduling via the HMO’s website and checking lab results	Do not intend to use	22 %	20 %	31 %
	Intend to use	70 %	72 %	61 %
	Do not have a clear stance in the matter	8 %	8 %	8 %
	The median score between 1–7	5.00	5.00	4.00
HMO’s website for consulting with specialist doctors, forums for medical information etc., via services based on chats, video chats etc.	Do not intend to use	52 %	54 %	41 %
	Intend to use	41 %	39 %	53 %
	Do not have a clear stance in the matter	7 %	7 %	6 %
	The median score between 1–7	1.00	1.00*	3.00
If you had the opportunity to use remote medical services, would you use them?	Do not intend to use	34 %	35 %	31 %
	Intend to use	60 %	59 %	65 %
	Do not have a clear stance in the matter	6 %	6 %	4 %
	The median score between 1–7	5.00	5.00	6.00

Mann–Whitney test was used to test the differences in the scores of the different groups

*p < 0.1

^a^On a scale of 1–7 (1 = Do not intend at all, 7 = very much intend)

The results shown in Table 2 indicate that most participants, Jews and non-Jews alike, intend to use their HMO website to obtain administrative information. Furthermore, most participants were willing to use remote medical services (60 %). The rate of non-Jewish participants that mentioned they intend to use forums and consult with doctors online was significantly higher in comparison to Jews (53 % as opposed to 39 %, respectively).

### Reasons for use/non-use of OHS

The results of the telephone survey indicate that 22 % of internet users do not use OHS at all. The main reasons reported for the lack of OHS use were the fact that OHS are not easy to use (32 %), there is “no need for OHS” (23 %) and a lack of awareness of these services (22 %). Furthermore, 10 % mentioned a “fear of technology” (ranked fourth). Additionally, examining the distribution of reasons by age revealed that the percentage of participants mentioning that OHS are not easy to use was relatively high among individuals aged 61+ and that the percentage of participants that mentioned “no need” was relatively high among individuals aged 45–60.

Among Internet users that do use OHS, the main reported reasons were that OHS use saves time and precludes the need to leave the house (53 %), facilitates easy and quick access to health services (39 %) and makes it easy to keep up with health-related subjects (19 %).

Among OHS users, 32 % reported that they receive some help from their family in using OHS. In the non-Jewish sector, the percentage of those who receive help from their family (45 %) is significantly higher than in the Jewish sector (30 %).

### Attitudes towards the use of OHS

Table 3 shows the mean values and standard deviations of the three categories of HIAM statements: barriers to OHS use, cues for action of use and perceived ease of use^5^. The findings indicate that for each type of OHS examined (using HMO website for administrative information and doctor consultation and using online forums for medical information), the mean values of statements in the categories of cues for action and perceived ease of use were significantly higher among OHS users than among non-users.

**Table 3:** Means and S.D. of the HIAM variables and attitudes by OHS use (among Internet users *N =* 414)

HIAM variables^a^		Use OHS for administrative information *N =* 276	Do not use OHS for administrative information *N =* 136	Use the Internet for medical consultation *N =* 70	Do not use the Internet for medical consultation *N =* 342	Use OHS for medical information *N =* 188	Do not use OHS for medical information *N =* 224
		Mean (standard deviation)					
Barriers	OHS are not precise and reliable enough	3.44 (2.33)	3.50 (2.38)	3.74 (2.41)	3.40 (2.33)	3.52 (2.33)	3.41 (2.36)
	OHS can breach the privacy of my medical information	3.42 (2.28)	3.62 (2.54)	3.56 (2.52)	3.46 (2.34)	3.36 (2.28)	3.59 (2.45)
	Technical and operational difficulties	2.75 (2.15)	3.13 (2.31)	2.87 (2.26)	2.87 (2.20)	2.57** (2.00)	3.14 (2.34)
Cues for action	My relatives encourage me to use OHS	3.36*** (2.62)	2.40 (2.28)	4.26*** (2.68)	2.81 (2.46)	3.60*** (2.67)	2.59 (2.36)
	The HMO’s advertisements encourage me to use OHS	4.05*** (2.50)	2.73 (2.27)	4.47*** (2.45)	3.44 (2.48)	4.22*** (2.53)	3.11 (2.37)
	The HMO’s staff’s advice to use OHS	3.77*** (2.49)	2.46 (2.19)	4.39*** (2.23)	3.13 (2.46)	3.82*** (2.52)	2.94 (2.35)
Perceived ease of use	Remote medical services will be easy and comfortable to use	5.50*** (1.85)	4.43 (2.48)	5.64** (1.83)	5.01 (2.21)	5.58*** (1.87)	4.72 (2.31)

^a^ The extent of agreement with the different statements on a scale of 1–7, 1 = do not agree at all, 7 = agree to a large extent

*p < 0.1, **p < 0.05, ***p < 0.01

Additionally, the findings show that the mean level of fear of technical and operational difficulties in using online forums to obtain medical information was significantly higher among participants who do not use forums than among those who use forums for medical information 6.

### Results of the analytical model: factors influencing extent of OHS use and intention to use OHS

Table 4 summarizes the results of the stepwise regressions analysis (OLS type) for two dependent variables: a) extent of use of HMO websites to obtain administrative information, and b) extent of use of HMO websites for doctor consultation and medical information. The scale for the dependent variables ranged from 1–6, where 1 = use very seldom and 6 = use very often. The regression analysis included only participants who use OHS.

**Table 4:** Regression analysis results of factors influencing the extent of use of different types of OHS

Dependent variable	The frequency of use of HMO’s website for^a^:			
	Administrative information *N* = 198 (R^2^ = 0.16***)		Consultation with doctors *N =* 47 (R^2^ = 0.34***)	
Explanatory variables				
	Beta coefficient	Std. Err	Beta coefficient	Std. Err
Age^b^	.001	.008	0.01	0.01
Gender (base = women)	0.32*	0.17	–0.05	0.28
Religion (base = non-Jew)	–0.22	0.27	0.61*	.320
perceived privacy of online use	0.03	0.05		
perceived ease of online use	0.16**	0.07	0.15**	0.07
perceived precision and reliability of online information	0.02	0.07	0.18**	0.08
fear from technical difficulties in online use	–0.04	0.04	-	-
Family’s encouragement for online use	0.09**	0.03	-	-
HMO’s advertisements on online use	0.03	0.04	-	-
HMO’s staff encouragement for online use	–0.02	0.04	-	-
concern with regard to health status (base = not concerned)	0.17**	0.07	-	-
The existence of long term care insurance (base = non)	0.16	0.18	-	-
Residence (base = lives alone)	–0.16	0.24	-	-
Constant	2.47***	0.73	1.21	0.93

*p < 0.1, **p < 0.05, ***p < 0.01

^a^Each of the two regressions have different set of explanatory variables, therefore there are missing rubrics in some places in the table

^b^Age is a continuous variable

The explanatory variables in the regressions of the two variables were age, gender (base = female), religion (base = non-Jew), HIAM categories (on a scale of 1–7 where 1 = do not agree at all and 7 = agree to a large extent), perceived ease of use of HMO website and extent of precision and reliability of online information. Furthermore, the regression of the extent of HMO website use for administrative information included additional HIAM variables: extent of perceived privacy of online use, extent of fear of technical difficulties in online use, extent of encouragement from family and HMO staff for online use, and influence of HMO advertisements in the media on online use. Additionally, the following variables were examined: extent of concern regarding health status (base = not concerned at all), existence of long-term care insurance (base = no insurance) and living alone or with family/other (base = lives alone)^7^.

The results in Table 4 (columns 2–3) show that after controlling for the rest of the explanatory variables, the following factors significantly influence extent of HMO websites use to obtain administrative information: a) Extent of perceived ease of online use; as perceived ease of use increased, so did extent of online use. b) Family encouragement for online use: As family encouragement of online use increased, the extent of this use was greater as well. c) Concern regarding health status; as concerns for health status increased, the extent of online use became greater as well. d) Gender: Men tended to use this type of online service more than women did. The rest of the factors examined did not significantly influence the dependent variable.

The results in Table 4 (columns 4–5) show that the following factors significantly influence extent of use of HMO websites for doctor consultation and forums for medical information: a) Extent of perceived ease of online use; as perceived ease of use increased, extent of online use increased as well. b) Extent of precision and reliability of online information; as perceived precision and reliability increased, extent of online use increased as well. c) Religion: Jews tended to use this type of online service more than non-Jews did. The results of an additional regression show that as the extent of perceived privacy decreases, so too does the extent of forum use on online websites (that are not the HMO website)^8^.

Table 5 summarizes the results of the regression with respect to factors influencing intention to use OHS among those who have access to the Internet. The OHS included the following three services (on a scale of 1–7, where 1 = do not intend at all and 7 = intend to a large extent): a) HMO website for administrative information; b) HMO website for doctor consultation; c) Remote medical services.

**Table 5:** Regression analysis results of the factors affecting the intention to use OHS

Dependent Variable	The extent of intention to use the HMO’s website for^a^:				The intention to use remote medical services *N =* 294 (R^2^ = 0.3***)	
	Administrative information *N =* 252 (R^2^ = 0.32***)		Doctor consultation *N =* 178 (R^2^ = 0.29***)			
Explanatory variables						
	Beta coefficient	Std. Err	Beta coefficient	Std. Err	Beta coefficient	Std. Err
Age^b^					–0.02*	0.01
perceived ease of online use	0.35***	0.08	0.35***	0.07	0.47***	0.06
Cues for action	0.18***	0.06	0.15*	0.08	0.13*	0.07
Uses the HMO’s website for administrative needs (base = does not use)	0.29***	0.06	0.72**	0.32	0.14	0.06
Concern with regard to health status (base = not concerned)	–0.68**	0.32				
Uses the HMO’s website for consultation and forums (base = does not use)	0.12**	0.05	0.86**	0.34		
Intends to use the HMO’s website for consultation (base = does not intend)					0.17***	0.06
Residence (base = lives alone)	0.34**	0.15				
Constant	0.48	0.64	0.55	0.39	1.97**	0.90

***p < 0.01, **p < 0.05, *p < 0.1

^a^Each of the two regressions have different set of explanatory variables, therefore there are missing rubrics in some places in the table

^b^Age is a continuous variable

The regression of intention to use HMO website for administrative information included the following explanatory variables: extent of concern with health status (base = not concerned), use of HMO website for consultation and forums (base = does not use), intention to use HMO website for consultation (base = does not intend), residence (base = lives alone), use of HMO website for administrative needs (base = does not use), and extent of perceived ease of online use and cues for action (including extent of encouragement for online use by family, HMO staff and advertisements).

The explanatory variables in the regression of intention to use the HMO website for consultation with specialists were: use of HMO website for administrative needs (base = does not use) and for consultation and forums (base = does not use), as well as perceived ease of online use and cues for action.

The explanatory variables in the regression of intention to use remote medical services were the use of the HMO website for consultation and forums (base = does not use), the intention to use the HMO website for consultation (base = does not intend), the use of the HMO website for administrative needs (base = does not use) and the perceived ease of online use and cues for action.

The results in Table 5 (columns 2–5) show that the extent of intention to use the HMO website for administrative information and for consultation with specialists during the coming year increases with greater perceived ease of use, more significant encouragement from family and HMO staff, more exposure to advertisements and more frequent use of the HMO website for administrative needs and consultation with specialists. Furthermore, the intention to use the HMO website for administrative information is greater when individuals are less concerned regarding their own health status and also for individuals who do not live alone.

Furthermore, the results in Table 5 (columns 6–7) show that the extent of intention to use remote medical services increases with higher perceived ease of use, more significant encouragement from family and HMO staff, greater exposure to advertisement and greater willingness to use the HMO website for consultation with specialists. Additionally, willingness to use remote medical services is greater among younger individuals.

## Discussion

The current research empirically examined the extent of use of OHS and remote medical services among the population of middle-aged to older adults in Israel. Furthermore, the research examined the main factors that influence the extent of OHS use among this population.

The results of a telephone survey of a national sample of 703 interviewees aged 45 and above show that about two third of the sample use the internet. Among internet users, the main use of OHS is via their HMO website in order to obtain administrative information. The second use of OHS was in consulting forums to obtain medical information, while less than twenty percent reported using their HMO website to consult with specialists.

Our findings reveal that 22 % of the internet users do not use OHS at all. The main reasons reported for lack of OHS use were the following: OHS are not easy to use, there is no need for OHS and people are unaware of these services. Indeed there is a documented gap between the skill and knowledge demands of OHS and user competencies to benefit from these tools [41]. Yet our results also show that most internet users, Jews and non-Jews alike, mentioned that they intend to use their HMO website for administrative information and that most were willing to use remote medical services.

Another result of the current research is that wealthier populations use some online services more frequently. For example, the rate at which internet users use their HMO website to obtain administrative information is higher among individuals with an education of 12+ years and among those with an average or higher income. Our results are compatible with Neter et al., [4] findings with respect to the Israeli population (18 years and older) that those who were highly eHealth literate tended to be younger and more educated than their less eHealth-literate counterparts. In addition, Choi and DiNitto [43] findings show that low-income older adults’ in the US have lower eHealth Literacy Scale score compared to the US population due to the lack of exposure to computer/Internet technology, lack of financial resources to obtain computers and technology, or medical conditions that restrict use.

Furthermore, we found that the rate of use of HMO websites to consult with specialists is higher among non-Jews than among Jews. Our results also indicate that the rate of use of forums to obtain medical information is higher among native Israelis, among individuals with an average or higher income and among women. Our result with respect to gender is compatible with the findings that being female in the U.S. was a consistent predictor of eHealth use across health care and user-generated content/sharing domains [2]. In addition, according to the Pew Internet and American Life Surveys about half of online women (52 %) say health and medicine is among the top three topics of interest to them, compared with 22 % among men [44].

In addition we found that individuals’ attitudes towards OHS use significantly influence their decision to use OHS. Specifically, OHS users perceived OHS as easier to use than did non-users. Furthermore, OHS users received more encouragement than non-users to use OHS from family members or the HMO staff or by being exposed to advertisements. Additionally, the extent of fear of operational difficulties involved in using online services to obtain medical information was higher among participants who do not use forums for medical information than among participants who use such forums. In fact, a recent study shows that the most prevalent concern raised by participants who communicated with a doctor about their online health information seeking related to the credibility or limitations in online information [45].

The results of the analytical model indicate that frequency of use of HMO websites to obtain administrative information increases with greater perceived ease of online use, more family encouragement for online use, concern for health status and among men.

Furthermore, the extent of HMO website use to consult with doctors and forums to obtain medical information increases with greater perceived ease of online use, higher precision and reliability of online services and among Jews. Yet, results of a recent study indicate that individuals with low health literacy (and related skills) have lower ability to evaluate online health information and have lower degree of trust in online health information [46].

Moreover, the extent of intention to use remote medical services is higher among younger individuals (probably due to their greater familiarity with forums and online interactions). Intention to use remote services is also greater when online use is perceived as being easier, when family members and HMO staff encourage online use, and when potential users are exposed to advertisements that encourage use.

Using OHS have many important benefits. Results of a study that compared the use of Information and Communications Technology (ICT) in health systems in Israel and Portugal showed that in both countries the increased deployment of ICT has furthered patient empowerment. The increased access of patients to web-based medical information can strengthen the role of patients in decision making and improve the physician-patient relationship [47, 48]. In addition, access and use of online health information in Israel provide an alternative/additional channel for information when e-patients consider health changes [49].

The current research adds to the existing literature by using an integrated model in the case of consumers that voluntarily use OHS systems to examine the intention to use OHS and the extent of actual use of OHS in Israel today. The results of the study not only indicate the factors affecting the intention and the usage of OHS but also map the gaps between socio- demographic groups in Israel (e.g., Jews and non- Jews). The results of the study may help to develop policy to enhance the use of OHS among the middle-aged to older adults in Israel in order to improve their quality of life and in order to save costs to the health system.

Yet the research reported here has some inherent limitations. The research questionnaire is based on participants’ reported answers with respect to their usage of the various OHS and intention to use OHS. Naturally, reported answers are not accurate variables and may be subject to variety of conscious and or unconscious psychological motivations of the self-reporting person. However, it is quite common method in the literature to elicit people actual action and intention (e.g., Huang [30]).

Another limitation of the study is the participants’ low response rate to the telephone survey. Perhaps those who respond more quickly and readily to phone surveys have systematically different approaches to the adoption of OHS, compared with those who tend not to participate in phone surveys. The distribution of the study sample according to socio-demographic characteristics was quite similar to that of the Israeli population (above 45 years old)^9^. However it is quite possible that even among people with similar socio-demographic characteristics, respondents are more likely to make use of OHS than non-respondents. Another limitation of the study is that it examined remote medical services that are in their incipient stages in Israel, and most people have little knowledge about them. Yet, it is important to study people’s opinions with respect to these types of services since they will become more and more accessible to the Israeli public in the near future.

Future research may examine the progress over time of usage and the intention to use all types of OHS including remote medical services. In addition, we hope that future research will examine changing attitudes and perceptions towards OHS in reference to the current study, which may be considered as a temporal benchmark of the present.

## Conclusions

Frequency of OHS use increases as the following factors increase: perceived ease of online use; extent of encouragement for online use; perceived reliability of online health services; and extent of advertisement exposure. In addition, OHS use is much more prevalent among wealthy populations.

Based on the findings of the study, we make the following recommendations to increase frequency of OHS use and to encourage more internet users to start using OHS: Extend and enhance advertisement in different media channels to emphasize and illustrate the worthwhileness of OHS use. Such advertisement should specifically emphasize online consultation with specialists via the HMO website, since this is the least common use of OHS today. The research reveals a major barrier with respect to online personal consultation. Therefore, advertisement should target this point in particular. Anther recommendation is to develop guidance programs for HMO staff (administrative and medical) to encourage patients to use OHS, since there is a documented gap between the skill and knowledge demands of eHealth systems and user competencies to benefit from these tools [50]. In addition, to develop and encourage programs that integrate students/pupils in exchange for scholarships. These students can provide personal guidance to older individuals regarding use of OHS websites. Finally, it is recommended to improve the user interface (display and operation) of OHS websites to make them more accessible to diverse populations (mainly older adults and those who do not speak Hebrew)^10^. The matter of system identification (user and password retrieval) is very important.

### Ethics approval and consent to participate

The ethics committee of the Max Stern Yezreel Valley College in Israel approved the current research (No. 2012–17).

### Availability of data and materials

The dataset supporting the conclusions of this article is available from authors upon request.

## Appendix 1

### A Survey concerning the use of OHS

#### Part A: Socio demographic and personal details

Age, marital status, education, nationality, immigration year, religious identification (1 = very religious, 5 = secular), income (from 1 = much above average to 5 = much below average), place of residence, HMO membership, supplementary insurance, private insurance, employment status, and individual’s self-evaluation of health status.

#### Part B—Accessibility, expertise, nature of use, and attitudes regarding information and communications technologies

I would like to ask you now a number of questions regarding computers, Smartphones, Tablets and the Internet.

- Do you own each one of these devices, and if so, do you use them to go online?
- Do you have a computer that is connected to the Internet, and if so, do you use it to go online?

- I do not have a computer that is connected to the Internet
- I have a computer that is connected to the Internet, but I do not use it to go online
- I use the Internet on the computer
  - Do you have a Smartphone that is connected to the Internet, and if so, do you use it to go online? (Including using Whatsapp, and different applications)
- I do not have a Smartphone that is connected to the Internet
- I have a Smartphone that is connected to the Internet, but I do not use it to go online.
- I use the Internet on the Smartphone (including applications like Whatsapp and Waze)
  - Do you have a Tablet computer that is connected to the Internet, and if so, do you use it to go online?
- I do not have a Tablet computer that is connected to the Internet
- I have a Tablet computer but I do not use it to go online
- I use the Internet on the Tablet computer
  - (For those who stated three times that they do not use the Internet) What is the main reason that you do not use the Internet today?
  - For those who use the Internet on more than on device: On which device do you use the Internet the most? The computer, 2. The Smartphone, 3. The Tablet computer

#### Part C—for those who use the Internet—Frequency of use, intention to use and use patterns of OHS

How often do you use each of the following OHS? On a the following scale: 1 = Do not use, 2 = Less than once a year, 3 = Once a year, 4 = Once every few months, 5 = Once a month, 6 = A number of times a month, 7 = Once a week or more.

- The HMO’s website for administrative information (such as: appointment scheduling and checking lab results via the website)
- The HMO’s website in order to consult with doctors, specialists etc.
- Forums in order to obtain medical information not related to the HMO.
  - To what extent do your friends/family assist you in using OHS? (on a scale of 1–7, 1 = not at all, 7 = very much).
  - For those who do not use OHS: State the main reasons for not using OHS
  - For those who use at least one OHS: To what extent do you intend to use the following OHS during the next year? On a scale of 1–7 (1 = do not intend at all, 7 = intend very much):
- The HMO’s website for administrative information (such as: appointment scheduling and checking lab results via the website)
- The HMO’s website in order to consult with doctors, specialists etc.
- If you had the option to use remote medical services (medical staff’s remote control on designated devices) would you use them?
  - To what extent do you feel that your privacy is protected when you use each one of the following OHS? On a scale of 1–7, (1 = not protected at all; 7 = very much protected):
- The HMO’s website for administrative information (such as: appointment scheduling and checking lab results via the website)
- The HMO’s website in order to consult with doctors, specialists etc.
- Remote medical services, when they will be available, such as: medical staff’s remote control on designated devices.
  - To what extent do you think that the information in each of the following online health services, is accurate and reliable enough: On a scale of 1–7 (1 = Not reliable and accurate at all; 7 = Very much reliable and accurate):
- The HMO’s website for administrative information (such as: appointment scheduling and checking lab results via the website)
- The HMO’s website in order to consult with doctors, specialists etc.
- Remote medical services, when they will be available, such as: medical staff’s remote control on designated devices.

#### Part D—Reasons for using OHS

- State the main reasons for using OHS

#### Part E—Attitudes regarding online health services

On a scale of 1–7 (1 = do not agree at all, 7 = very much agree), to what extent do you agree with the following statements?

- (For those who use OHS) HMO’s website for administrative information, such as appointment scheduling via the website, is easy and comfortable to use
- (For those who use OHS) Using HMO’s website in order to consult with doctors, specialists, receive medical information etc., is easy and comfortable for me
- (For everyone)When remote medical services, like a medical staff’s remote control on designated devices will be available, it will be easy and comfortable for me to use them.
- (For everyone) I am worried that using OHS will not be accurate and reliable enough
- (For everyone) I am worried that OHS are not secure enough, and might breach the privacy of my medical information
- (For everyone) I am worried regarding the technical and operational difficulties I will have while using OHS
- (For everyone) My family and/or relatives and friends encourage me to use OHS
- (For everyone) The HMO’s website advertising, encourage me to use OHS
- (For everyone) The HMO’s medical and/or administrative staff encourage me to use OHS

## Appendix 2

**Table 6:** The socio- demographic characteristics of the sample and the population in Israel (45 years and above)

		Sample distribution (*N =* 703)	Population distribution
All		100 %	100 %
Gender	Women	58 %	53 %
	Men	42 %	47 %
Age	45–59	48 %	50 %
	60–64	16 %	15 %
	65+	36 %	35 %
Religion	Jews	82 %	83 %
	Other	18 %	17 %
Education	12 and less	44 %	37 %
	More than 12	56 %	63 %

## Abbreviations

EHR: electronic health record
HBM: the health belief model
HIAM: healthcare information adoption model
HMO: health maintenance organizations
OHS: online health services
PHR: personal health record
TAM: the technology acceptance model
UTAUT: unified theory of acceptance of technology model

## Footnotes

1 The cultural characteristics of Jews and Arabs differ significantly. Arab society has been characterized as relatively traditional, and collectivist-oriented [51]. Oyserman [52] found that Israeli Arabs are characterized by greater collectivism than are Israeli Jews.

2 The sample size was calculated according to Whitehead’s [53] approach, while considering the nature of dependent and independent variables, and for OR = 1.3, power = 0.8, alpha = 0.05.

3 CBS, Localities in Israel, http://www.cbs.gov.il/ishuvim/ishuvim_main.htm

4 The firm conducted the telephone survey five days a week, at different hours and on different days. For the purpose of controlling and extracting the sample, the names of respondents who were not interviewed were recorded on a special list that controlled repeated trials (up to four repetitions per household) on different days and hours. In other cases repeated trials were conducted at specific dates determined in accordance with the interviewees. In addition, the firm conducted a persuasion conversation with those who refused to participate in the survey

5 With respect to the other HIAM categories, no significant differences were found between OHS users and non-users.

6 With respect to the other barrier category items, no significant difference was found between OHS users and non-users.

7 The explanatory variables which relate to the extent of use of HMO websites to obtain administrative information, are different from the explanatory variables which relate to the extent of use of HMO websites for doctor consultation and medical information on the web. For example : the extent of fear of technical difficulties in online use and the HMO advertisements in the media on online use are relevant to the use of HMO websites to obtain administrative information, but are less relevant for searching medical information on the web.

8 The results are not shown in a table.

9 See Table 6 in Appendix 2

10 The majority of HMO sites are available in Hebrew with limited information in English. Most of the sites are not available in Arabic, or Russian.
